# Exercise-Induced Bronchoconstriction Identified Using the Forced Oscillation Technique

**DOI:** 10.3389/fphys.2019.01411

**Published:** 2019-11-15

**Authors:** Leigh M. Seccombe, Matthew J. Peters, Lachlan Buddle, Claude S. Farah

**Affiliations:** ^1^Thoracic Medicine, Concord Hospital, Sydney, NSW, Australia; ^2^Faculty of Medicine and Health, Sydney University, Camperdown, NSW, Australia; ^3^The Woolcock Institute of Medical Research, Sydney, NSW, Australia

**Keywords:** airway resistance, exercise-induced bronchoconstriction, airway reactance, asthma, respiratory function

## Abstract

**Objective:** Lung mechanics using the forced oscillation technique (FOT) is suggested to be equivalent and more sensitive in determining exercise-induced bronchoconstriction (EIB) than spirometry. Dynamic alterations in minute ventilation (V_E_) may affect this measurement. We investigated changes in FOT parameters post exercise challenge (EC) in people with asthma as compared to spirometry. The rate of recovery and any effect of raised V_E_ following exercise on FOT parameters were also assessed.

**Method:** Airway resistance (R_5_) and reactance (X_5_) at 5 Hz and V_E_ were measured prior to forced expiratory volume in 1 s (FEV_1_) before and up to 20 min after a standard EC in people with asthma and healthy controls. Airway hyperresponsiveness to the hyperosmolar mannitol test was measured in the asthmatic subjects within 1 week of the EC. Baseline and sequential measures were assessed using repeated measures ANOVA and Pearson’s correlation. Group demographics and recovery data were compared using an unpaired *t* test.

**Results:** Subjects with current asthma (*n* = 19, mean ± SD age 28 ± 6 years) and controls (*n* = 10, 31 ± 5 years) were studied. Baseline FEV_1_, R_5_, X_5_, and V_E_ were similar between groups (*p* > 0.09). Airway hyperresponsiveness was present in 12/19 asthmatic subjects. The EC max % change of R_5_ and X_5_ correlated with FEV_1_ (*r* > 0.90) and were only different to controls in those with asthma that responded by FEV_1_ criteria (*p* < 0.01). EC recovery of R_5_ was similar to FEV_1_; however, X_5_ was greater (*p* = 0.03). Elevated V_E_ post EC did not affect the % change in FOT parameters across all subjects (*p* > 0.3). R_5_ and X_5_ were highly sensitive in determining a positive EC response (80–86%), but X_5_ was more specific (93 vs. 80%).

**Conclusion:** FOT parameters tracked with forced maneuvers and were not influenced by increased ventilation following an exercise challenge designed to elicit EIB. FOT identified EIB similarly to spirometry in patients with asthma.

## Introduction

Exercise-induced bronchoconstriction (EIB) describes increased sensitivity to exercise stimuli that cause airway narrowing *via* airway smooth muscle contraction (Joos et al., 2003). Commonly associated with asthma, the presence and degree of EIB can be assessed in the laboratory using an exercise challenge test (EC), a stimulus that releases inflammatory mediators (Anderson et al., 1982; Weiler et al., 2016) *via* osmotic and thermal effects of respiratory water loss (Anderson et al., 1982).

Traditionally an EC is assessed using forced expiratory volume in 1 s (FEV_1_), where *a* ≥ 10% decline from baseline is considered positive (Parsons et al., 2013). Measurement is dependent on the patient’s ability to perform maximal and repeatable efforts, which may be challenging for some and may independently alter airway resistance. The required deep inhalation is known to alter airway caliber in those with airway hyperresponsiveness, affecting airway resistance and therefore the sensitivity of FEV_1_ to detect EIB. In addition, other lung volume-related responses (such as hyperinflation) are not detectable by spirometry.

The use of oscillometry methods such as the forced oscillation technique (FOT) or the impulse oscillometry system can measure total respiratory system impedance encompassing the resistive, elastic, and inertial properties of the airways (Oostveen et al., 2003). Increased airway resistance and more negative reactance have been identified in those with airway obstruction defined by spirometry and provide an insight into the behavior of the airway tree such as reduced airway caliber and increased ventilation inhomogeneity. A previous study using the impulse oscillometry system during EC in adults with probable EIB (Evans et al., 2005) established the technique as repeatable, clinically relevant and to correlate with spirometry. FOT is performed during tidal breathing, which eliminates the need for forced maximal maneuvers required during spirometry. As such, the test is well tolerated by patients and suitable for serial measurement as required during a challenge test. The increasing availability of commercial FOT devices highlights the need to define the utility and any limitation to the adoption of this lung function modality in the clinical setting.

The impact of the increased minute ventilation (V_E_) and any change in the operating lung volume on FOT parameters during the challenge test has not been previously studied. Such patient factors could affect the reliability of the test. Dynamic hyperinflation has been previously described in both healthy subjects and those with airway hyperresponsiveness (Anderson et al., 1972). It is also known that FOT parameters are dependent on lung volume when measured in a resting steady state condition (Oostveen et al., 2003; Milne et al., 2019). As such, clinical practice recommendations suggest that measurement should be performed with the patient breathing at functional residual capacity. A stable respiratory rate, and consequently V_E_, is also recommended due to concern that an increased respiratory rate could affect signal-to-noise. Both of these factors, however, cannot be controlled following an exercise test since the test is associated with increased V_E_ and measurement is inherently not performed during steady state.

We hypothesized that measures of airway mechanics using FOT are sensitive in detecting EIB in comparison to forced maneuvers and are not confounded by the post exercise increase in V_E_. The aim of this investigation was (1) to compare changes in FOT and spirometry parameters post EC in people with asthma and healthy controls and (2) to investigate the rate of recovery and any effect of raised V_E_ following exercise on FOT parameters.

## Methods

### Subjects and Study Design

This was a prospective study that was reviewed and approved by the Sydney Local Health District Human Ethics Review Board (HREC/15/CRGH/266, NSW, Australia) with written informed consent obtained from all subjects in accordance with the Declaration of Helsinki. Some data have been previously published in part (Seccombe et al., 2018).

Subjects with asthma were recruited from a hospital-based airways disease clinic. A standard definition of current asthma was used; prior clinician diagnosis of asthma and symptoms or use of asthma medications within the last 12 months was used. All subjects were not permitted to have an upper respiratory tract infection within the past 4 weeks, any known cardiac disease or claustrophobia. Prior to all tests, subjects were asked to abstain from caffeine and exercise for at least 4 h, long-acting beta-agonists and/or inhaled corticosteroids for at least 48 h, tiotropium bromide or antihistamines for at least 72 h, and short-acting beta agonists for at least 12 h (Anderson and Kippelen, 2013). Healthy age matched controls (±1 SD of the mean age of the study group) who had no history of lung or cardiac disease were recruited for comparison *via* community advertisement.

At the initial visit, all subjects with asthma performed a mannitol challenge to determine the presence of airway hyperresponsiveness. At the second visit (which was within 1 week of the first visit), subjects sequentially completed the asthma control test, exhaled nitric oxide measurement and an exercise challenge designed to elicit EIB (EC). All tests were performed by experienced Scientific Officers in an accredited laboratory. All lung function measurement devices were successfully calibrated incorporating ambient conditions prior to each test.

### Experimental Procedures

#### Mannitol Challenge

A mannitol challenge (Aridol™, Pharmaxis, Frenchs Forest, NSW, Australia) was performed according to Anderson et al. (1997). The dose protocol consisted of 0, 5, 10, 20, 40, 80, 160, 160, and 160 mg mannitol delivered *via* osmohaler. Three FEV_1_ maneuvers were performed 60 s after each dose, and the highest was compared to that measured after the 0-mg capsule to calculate the percent decrease. The challenge was terminated when a 15% decrease in FEV_1_ was measured or a total cumulative dose of 635 mg had been given. Airway hyperresponsiveness to mannitol was defined as a 15% fall in FEV_1_ to a provoking cumulative dose of 635 mg or less (PD_15_).

#### Asthma Control Test

Asthma control was determined using the patient-based five-item Asthma Control Test survey (Nathan et al., 2004). The subject completed the survey independently from the Scientific Officer, a score ≥ 20 indicates well controlled asthma.

#### Exhaled Nitric Oxide

Exhaled nitric oxide was measured (CLD-88sp, Ecomedics, Dürnten, Switzerland) before any forced respiratory maneuvers according to consensus guidelines (American Thoracic Society and European Respiratory Society, 2005). Following a full inhalation of zero nitric oxide (scrubbed) room air, the patient was instructed to exhale at 50 ± 5 ml s^−1^ until a plateau was measured. The recorded value was the mean of two values within 5% or mean of three values within 10% of each other. A value >25 ppb was regarded as elevated.

#### Exercise Challenge Test

An EC designed to elicit EIB was performed according to American Thoracic Society recommendations (Parsons et al., 2013). Subjects breathed room air on a cycle ergometer (VIAsprint 150p, Ergoline, Bitz, Germany) with a nose clip *in situ*. Continuous breath-by-breath gas analysis was measured, while the subject breathed through a mouthpiece connected to a metabolic cart (Oxycon Pro, Jaeger, Hoechberg, Germany). V_E_ was continuously monitored to ensure workload was titrated to elicit a target greater than 60% of the subjects estimated maximal voluntary ventilation (calculated as baseline FEV_1_ × 40) (Anderson and Kippelen, 2013; Parsons et al., 2013). The target workload was estimated using a validated algorithm and ramped over 3–4 min to achieve the target V_E_ for more than 4–5 min (Crapo et al., 2000).

### Lung Function Measurements

FOT and then spirometry were performed prior to (baseline) and at 3-, 5-, 10-, 15- and 20-min post exercise.

#### Forced Oscillation Technique

Respiratory system impedance to derive resistance (R_5_) and reactance (X_5_) at 5 Hz was assessed using the standardized FOT recommendations (Oostveen et al., 2003) (tremoFlo software build 1.0.40.38, Thorasys, Montreal, Canada). Measurements were collected during 30-s of tidal breathing with the subject seated upright and with cheeks supported. Acceptability included at least three breaths free from artifact due to occlusion, leak or drift, or extreme (>5 SD of mean) or negative resistance. The mean value generated from each measurement by the software was recorded.

#### Spirometry

Spirometry to derive FEV_1_ and forced vital capacity was measured according to American Thoracic Society/European Respiratory society guidelines (Miller et al., 2005) (Easy on-PC, ndd Medical Technologies, Zurich, Switzerland). The highest values of two repeatable, from three acceptable, efforts were recorded. Reference values were derived from the Global Lung Initiative (Quanjer et al., 2012).

#### Data Analysis

The primary measures of lung function were R_5_, X_5_, and FEV_1_ collected at each time point. EC response was expressed as the maximum fall in these values from baseline following exercise as a percentage of the baseline (pre-exercise) value. A ≥10% fall in FEV_1_ was considered positive (Parsons et al., 2013) and defined as “EC-positive.” The values at the 20-min time point following EC as a percentage of the baseline were used to assess and compare the rate of recovery. At each time point, V_E_ was calculated from the product of mean tidal volume and the mean respiratory rate measured during the 30 s FOT recording.

### Statistical Analysis

Specialist statistical advice was received. Statistical analysis was performed using SPSS Statistics version 25 (IBM Corporation, Armonk, NY, USA) and graphs prepared using GraphPad Prism 7 (GraphPad Software Inc., La Jolla, California, USA). Values for FEV_1_ post exercise that remained higher than the pre-exercise value were censored as 0% fall and sequential FEV_1_, R_5_, X_5_, and V_E_ post EC as % change from baseline was compared across groups using a repeated measures ANOVA. A one-tailed Pearson’s correlation investigated any association between R_5_, X_5_, FEV_1_, and V_E_ (including subdivisions of tidal volume and respiratory rate). Receiver operating characteristic curves were used to detect sensitivity and specificity for detecting a positive response to the EC as determined by FEV_1_ using FOT parameters. Group demographics, EC% maximum change, and recovery data were compared using an unpaired *t* test. Data are expressed as mean ± SD unless otherwise stated. A *p* < 0.05 was considered significant.

## Results

### Subjects and Baseline Lung Function Characteristics

Nineteen subjects with current asthma and 10 controls were studied. There were no differences in baseline demographics between groups (Table 1). Most patients with asthma were mildly symptomatic with an ACT score of 19.7 ± 4.3 and had elevated exhaled nitric oxide (52 ± 46 ppb) as compared to controls (21 ± 13 ppb) that did not reach significance (*p* = 0.06) (Table 2). At baseline, two thirds (12/19) of subjects with asthma had a normal FEV_1_/forced vital capacity ratio (above the lower limit of normal), and only one was mildly obstructed with a FEV_1_ below 80% of predicted. Two thirds (12/19) of subjects demonstrated airway hyperresponsiveness to mannitol with a PD_20FEV1_M of 165 ± 129 mg.

**Table 1:** Subject baseline characteristics.

Category	Asthma	Healthy normal
Male:female	8:11	5:5
Age (years)	28 ± 6	31 ± 5
Height (cm)	169 ± 11	170 ± 9
BMI (kg/cm^2^)	23.8 ± 3.1	24.0 ± 2.9
FEV_1_ (L)	3.46 ± 0.75	3.51 ± 0.65
FEV_1_ (%predicted)	93 ± 11	99 ± 13
FEV_1_/FVC ratio	0.77 ± 0.07*	0.85 ± 0.04
R_5_ (cm H_2_O s L^−1^)	3.49 ± 1.21	2.90 ± 1.02
X_5_ (cm H_2_O s L^−1^)	−1.20 ± 0.50	−1.00 ± 0.35

*Data are mean ± SD. BMI, body mass index; FEV_1_, forced expiratory volume in 1 s; FVC, forced vital capacity; R_5_, resistance at 5 Hz; X_5_, reactance at 5 Hz*.

* *Vs. control (*p* < 0.02), unpaired *t* test*.

**Table 2:** Lung function and exercise response.

	Asthma		Controls
Category	EC positive	EC negative	
Subject (*n*)	5	14	10
ACT score	18 ± 6	20 ± 3	—
eNO (ppb)	73 ± 46^*^	43 ± 45	21 ± 13
Mannitol positive	100%	50%	—
PD_15_M	128 ± 101	213 ± 136	—
FEV_1_ (%max change from baseline)	20 ± 13*,^†^	4 ± 3^*^	1 ± 1
R_5_ (%max change from baseline)	82 ± 105^*^^,^^†^	16 ± 16	5 ± 10
X_5_ (absolute max change from baseline)	1.76 ± 1.71^*^^,^^†^	0.11 ± 0.26	0.14 ± 0.22
***Exercise response***			
Watts	126 ± 40	108 ± 36	113 ± 22
V_E_ (L min^−1^)	89 ± 23	78 ± 13	78 ± 11
%max predicted HR	88 ± 4	88 ± 5	87 ± 4
***Post exercise response***			
Max V_E_			
L min^−1^	25 ± 9	26 ± 7	23 ± 8
%change from baseline	109 ± 68	101 ± 42	97 ± 70
Max RR			
bpm	9 ± 4	9 ± 1	9 ± 3
%change from baseline	17 ± 25	28 ± 26	36 ± 42
Max V_T_			
L	1.78 ± 0.57	1.65 ± 0.61	1.43 ± 0.70
% change from baseline	97 ± 53	76 ± 44	55 ± 33

*Data are mean ± SD*.

*EC, exercise challenge; ACT, asthma control test; eNO, exhaled nitric oxide; PD_15_M, provoking cumulative Mannitol dose for a 15% fall in FEV_1_; FEV_1_, forced expiratory volume in 1 s; FVC, forced vital capacity; R_5_, resistance at 5 Hz; X_5_, reactance at 5 Hz; V_E_, minute ventilation; HR, heart rate; RR, respiratory rate; V_T_, tidal volume*.

* *Vs. control*.

† *Vs. EC negative, unpaired *t* test, *p* < 0.02*.

### Exercise Challenge Test

Laboratory ambient conditions were 49 ± 12% relative humidity and 22 ± 1°C. Average V_E_ measured during the EC was 81 ± 15 L min^−1^ that equated with 59 ± 6% of the estimated maximal voluntary ventilation, corresponding with an average heart rate of 87 ± 4% predicted maximum. There was no difference between groups in the exercise response, including ventilatory parameters during or following exercise (Table 2).

**Figure 1:**
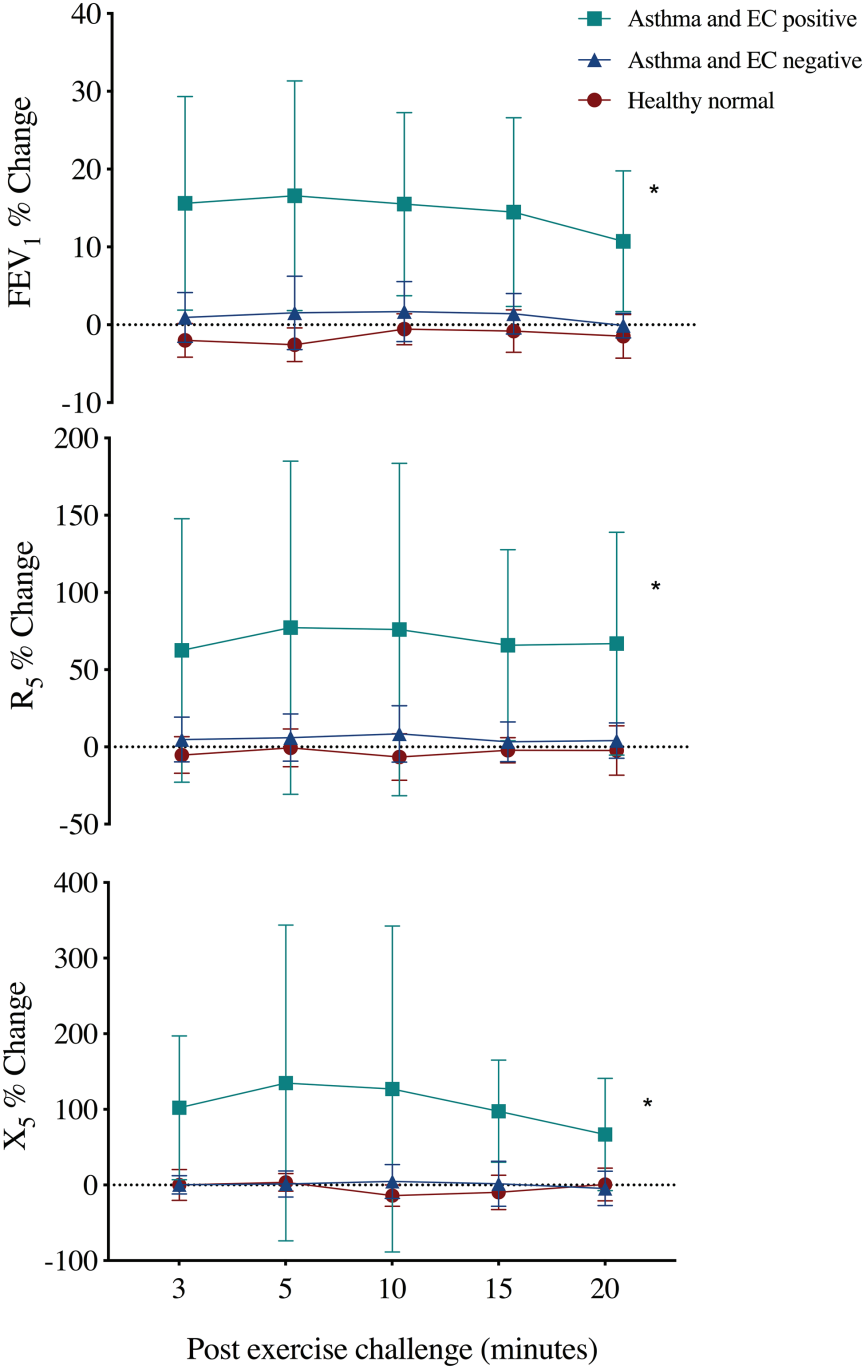
Mean ± SD percent change from baseline in forced expiratory volume in 1 s (FEV_1_), respiratory resistance (R_5_), and reactance (X_5_) at 5 Hz following an exercise challenge test in 19 subjects with current asthma that responded by FEV_1_ criteria (EC positive asthma, *n* = 5), did not respond (EC negative asthma, *n* = 14) and 10 healthy normals. *Repeated measures ANOVA, *p* < 0.02.

Five of the 19 subjects with asthma were EC positive. The R_5_ and X_5_ responses following EC were different in this group (max %change 92 ± 100% and 178 ± 188%) as compared to EC-negative asthma and healthy normal subjects (max %change 7 ± 7% and 16 ± 14%, *p* < 0.02) (Figure 1). The % recovery to baseline from maximum change at 20 min post EC was greater for X_5_ (*p* = 0.03) but similar between R_5_ and FEV_1_ (65 ± 21%, 43 ± 35%, and 26 ± 16%, respectively). Following EC, max % change FEV_1_ correlated with R_5_ (*r* = 0.92, *p* = 0.001) and X_5_ (*r* = 0.91, *p* = 0.001) across all subjects.

**Figure 2:**
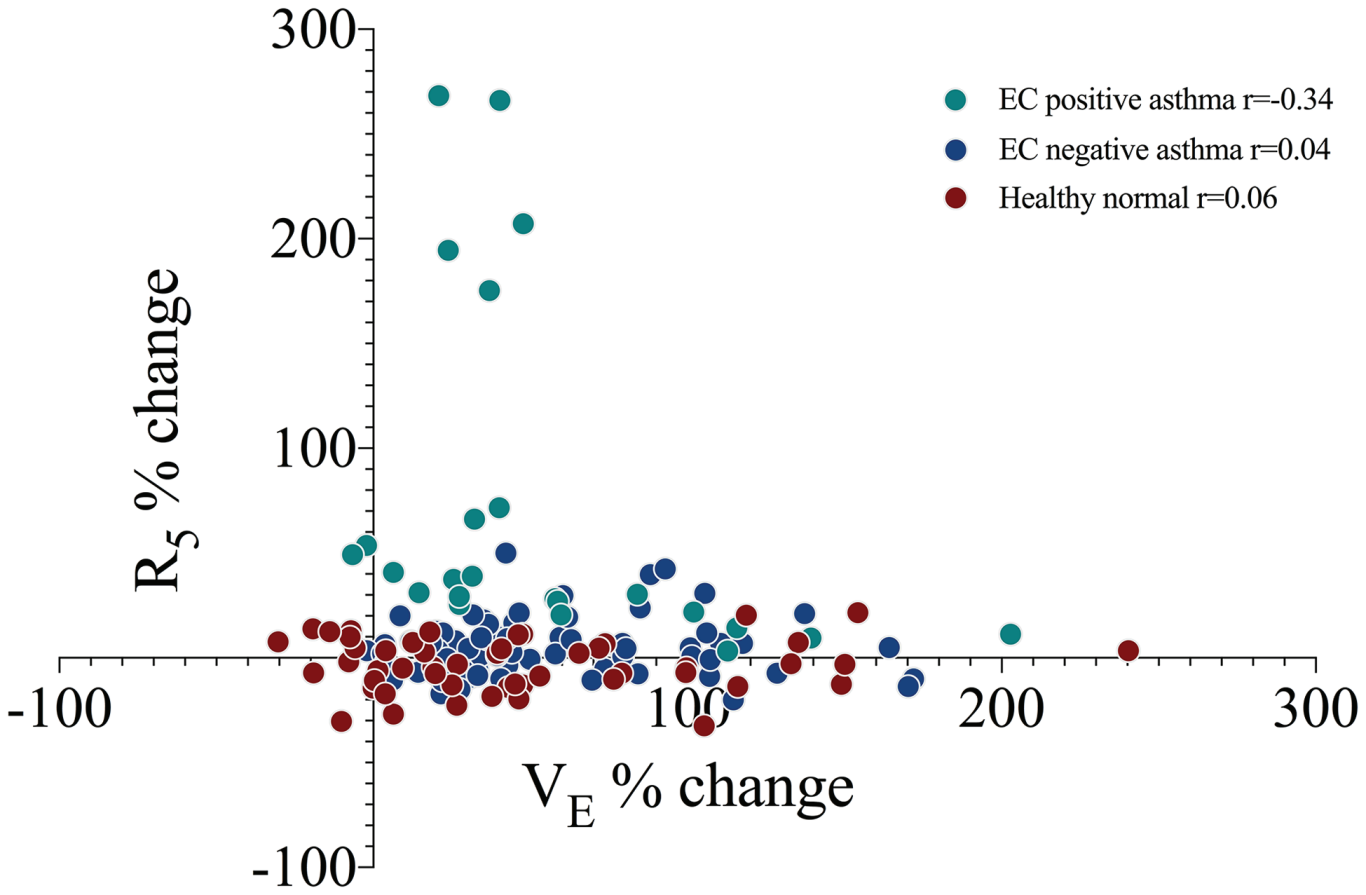
The correlation between minute ventilation (V_E_) and airway resistance at 5 Hz (R_5_) as a percent change from baseline at four sequential time points following an exercise challenge in 19 subjects with current asthma that responded by spirometry criteria (EC positive asthma, *n* = 5), did not respond (EC negative asthma, *n* = 14) and 10 healthy normals. *r*, Pearson’s correlation.

V_E_ was significantly elevated post EC (24.6 ± 7.3 L) as compared to baseline (12.6 ± 3.5 L, *p* = 0.0001), with a max change of 101 ± 55%. This was *via* a significant increase in both tidal volume (72 ± 43% max change) and respiratory rate (29 ± 32% max change) (*p* < 0.0001). The change in ventilatory parameters did not relate to the % change R_5_ (Figure 2) or X_5_ across all subjects or in those that were EC positive (*r* < 0.4, *p* > 0.1). The ventilatory responses during and following exercise were similar between groups (Table 2).

**Figure 3:**
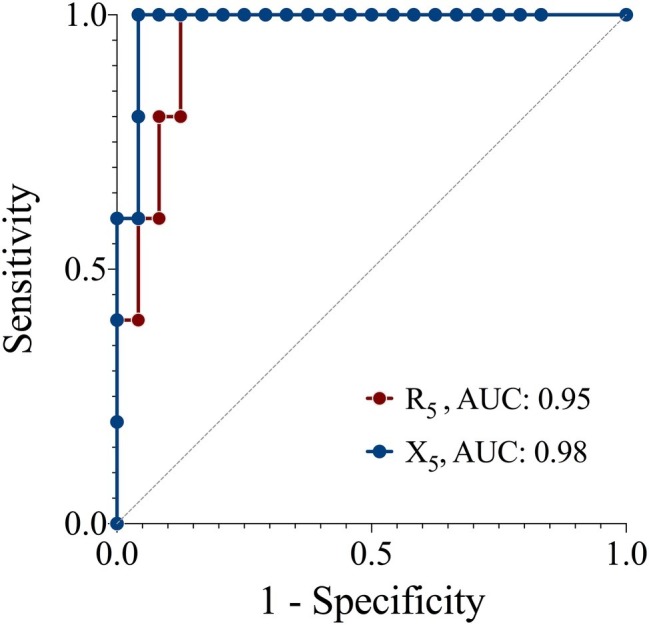
Receiver operator characteristic curves of the sensitivity and specificity of resistance (R_5_) and reactance (X_5_) at 5 z for detecting a positive response to an exercise challenge by spirometry criteria. Reference (solid line), AUC, area under the curve.

The sensitivity and specificity of R_5_ and X_5_ to detect a positive response to the EC as determined by FEV_1_ are presented in Figure 3. R_5_ had a sensitivity and specificity of 80 and 86%, X_5_ had a sensitivity and specificity of 80 and 93%, respectively. A cut-off of 27% increase in R_5_ and 47% decrease in X_5_ yielded optimal sensitivity and specificity for a positive response as detected using FEV_1_.

## Discussion

In patients with asthma, measurement of respiratory system impedance using the FOT identified EIB during an exercise challenge when compared to the accepted FEV_1_ criteria. Furthermore, the change in resistance and reactance was not unduly influenced by the change in V_E_ or respiratory rate following exercise. The results support the measurement of EIB in the laboratory using FOT as an alternative to spirometry.

The potential effect of raised respiratory rate or V_E_ post exercise has not been previously addressed. Standard FOT measurement requires quiet spontaneous resting breathing at functional residual capacity (Oostveen et al., 2003), yet variable changes in this value post exercise and/or due to bronchoconstriction can potentially alter measures of impedance (Anderson et al., 1972; van den Elshout et al., 1990). A single previous publication reported similar strong agreement between spirometry and impedance measured with impulse oscillometry system in an uncontrolled group of adult subjects with probable EIB (Evans et al., 2005). We felt it important to determine any influence of alterations in V_E_, either *via* respiratory rate or tidal volume post EC, on repeated FOT measures. V_E_ remained highly elevated following exercise during the data collection period, predominately from an expansion of tidal volume. This was similarly seen across groups of those with responsive asthma, non-responsive asthma and healthy controls. Reassuringly, there was no relationship between the FOT parameters of interest and V_E_, respiratory rate or tidal volume.

FOT is highly sensitive to changes in airway function during an exercise challenge test. The change in resistance and reactance following the EC predictably tracked with the change in FEV_1_. There was a clear separation in these measurements between subjects that were responsive according to the accepted FEV_1_ cut-off as compared to non-responsive asthma and control subjects. Indeed, both R_5_ and X_5_ were highly sensitive and specific for a positive EC. The optimal derived cut-offs for R_5_ and X_5_ to detect a 10% fall in FEV_1_ were 27 and 47%, respectively. Interestingly, these bronchoconstrictor cut-off values are similar in magnitude to previously published cut-offs for bronchodilator reversibility (32% change for R_5_ and 44% change for X_5_) (Oostveen et al., 2013).

The observation that changes in resistance related closely to changes in FEV_1_ is physiologically consistent since both measurements are influenced by airway caliber. Reactance, on the other hand, relates to the stiffness of the respiratory system as measured during tidal breathing and is dependent on the volume of accessible lung during the measurement (Milne et al., 2019). The development of bronchoconstriction during a challenge test results in a more heterogeneous lung with physiologically different lung units and more disparate time constants contributing to the overall impedance measured at the mouth. Hence, the more negative reactance that develops during challenge tests is likely a reflection of the narrowing and/or airway closure that develops in the distal airways.

The change in reactance and resistance during a challenge test provides physiologically relevant insights into airway behavior. It is noteworthy that reactance recovered faster than resistance or FEV_1_ post exercise. We hypothesize that this early recovery in X_5_ may reflect early functional recovery in the distal airway compartment before a measured improvement is seen in the indices that reflect more proximal airway caliber. The exact mechanisms are beyond the scope of this study but likely reflect a complex interaction of changes in the small airway caliber, altered transmural forces, and parenchymal tethering. Data collection in this study was terminated at 20-min post EC as recommended by clinical guidelines. Though not the primary objective of this study, a longer period may have allowed for further analysis of the time-dependent recovery of the various parameters.

Reassuringly, all subjects with a positive EC response using FEV_1_ criteria also exhibited a change using FOT. This indicates that FOT measurement can be safely performed during these tests without concerns for unrecognized bronchoconstriction.

There are some limitations to our study. Large variability was seen in the EC responders in R_5_ and X_5_; however, this was similarly observed with FEV_1_, and the changes were strongly correlated. As the EC is not a “dose response” challenge, inter-subject variability in airway response across a broad selection of subjects with “current asthma” is not unexpected (Anderson et al., 2010). The higher than ideal ambient humidity of the laboratory, though still within the acceptable range (Crapo et al., 2000), may have contributed to an underestimation of EIB. Nevertheless, this should not confound the current results since the analysis compared the various lung function parameters during the EC irrespective of the severity of EIB. The maximal inhalation required prior to the measurement of FEV_1_ can alter airway resistance, and it follows that FOT measured during tidal breathing may be more sensitive in detecting EIB. While FOT was measured prior to spirometry at all time points, it is important to repeat this study with and without FEV_1_ measurement as a validation study to determine repeatability of the test and confirm cut-off thresholds. Our cohort has small subject numbers, but the results are novel using a commercially available FOT device and suggest that FOT may be a clinically appropriate lung function measurement during a challenge test. Clearly, there is a need to derive clinically relevant cut points for more widespread uptake of FOT during challenge testing.

In conclusion, our results show that FOT measures detected bronchial reactivity to exercise as defined by a change in FEV_1_. Importantly, the FOT values are not confounded acutely by the raised minute ventilation post exercise. Hence, FOT measurement may be an alternative lung function test for the assessment of exercise-induced bronchoconstriction. It could be most useful in subjects who cannot perform spirometry to a satisfactory standard in the period after exercise or in whom deep breath-induced bronchoconstriction or bronchodilatation is problematic.

## Data Availability Statement

The datasets generated for this study are available on request to the corresponding author.

## Ethics Statement

The studies involving human participants were reviewed and approved by Sydney Local Health District Human Ethics Review Board. The patients/participants provided their written informed consent to participate in this study.

## Author Contributions

LS and CF contributed conception and design of the study. LS and LB performed data collection. LS organized the database and performed the statistical analysis. LS and CF wrote the first draft of the manuscript. All authors contributed to manuscript revision, read and approved the submitted version.

### Conflict of Interest

The authors declare that the research was conducted in the absence of any commercial or financial relationships that could be construed as a potential conflict of interest.

## Abbreviations

EIB: exercise-induced bronchoconstriction
EC: exercise challenge test
FEV_1_: forced expiratory volume in 1 s
FOT: forced oscillation technique
R_5_: resistance at 5 Hz
V_E_: minute ventilation
X_5_: reactance at 5 Hz.
